# Model Specification and the Reliability of fMRI Results: Implications for Longitudinal Neuroimaging Studies in Psychiatry

**DOI:** 10.1371/journal.pone.0105169

**Published:** 2014-08-28

**Authors:** Jay C. Fournier, Henry W. Chase, Jorge Almeida, Mary L. Phillips

**Affiliations:** 1 Department of Psychiatry, University of Pittsburgh School of Medicine, Pittsburgh, Pennsylvania, United States of America; 2 Department of Psychiatry and Human Behavior, The Warren Alpert Medical School of Brown University, Providence, Rhode Island, United States of America; King’s College London, United Kingdom

## Abstract

Functional Magnetic Resonance Imagine (fMRI) is an important assessment tool in longitudinal studies of mental illness and its treatment. Understanding the psychometric properties of fMRI-based metrics, and the factors that influence them, will be critical for properly interpreting the results of these efforts. The current study examined whether the choice among alternative model specifications affects estimates of test-retest reliability in key emotion processing regions across a 6-month interval. Subjects (N = 46) performed an emotional-faces paradigm during fMRI in which neutral faces dynamically morphed into one of four emotional faces. Median voxelwise intraclass correlation coefficients (mvICCs) were calculated to examine stability over time in regions showing task-related activity as well as in bilateral amygdala. Four modeling choices were evaluated: a default model that used the canonical hemodynamic response function (HRF), a flexible HRF model that included additional basis functions, a modified CompCor (mCompCor) model that added corrections for physiological noise in the global signal, and a final model that combined the flexible HRF and mCompCor models. Model residuals were examined to determine the degree to which each pipeline met modeling assumptions. Results indicated that the choice of modeling approaches impacts both the degree to which model assumptions are met and estimates of test-retest reliability. ICC estimates in the visual cortex increased from poor (mvICC = 0.31) in the default pipeline to fair (mvICC = 0.45) in the full alternative pipeline – an increase of 45%. In nearly all tests, the models with the fewest assumption violations generated the highest ICC estimates. Implications for longitudinal treatment studies that utilize fMRI are discussed.

## Introduction

Functional neuroimaging holds tremendous promise for advancing our understanding of both healthy psychological processes and those processes that underlie the development and maintenance of mental illness. The identification of neuroimaging-derived biomarkers of psychopathology could allow us to develop more effective, individualized treatment approaches that target the specific pathologies present in individual patients. For such a research program to succeed, however, it is critical that the validity and reliability of the metrics derived from functional neuroimaging be firmly established. In this study, we examine the degree to which the choice of modeling parameters can affect estimates of test-retest reliability. Specifically, we hypothesize that altering one’s modeling pipeline to account for additional sources of structured noise can affect the degree to which the assumptions of the underlying statistical model are met and can improve estimates of test-retest reliability.

### Test-Retest Reliability in fMRI

The importance of utilizing reliable research instruments in science cannot be overstated (see [1] for a discussion of psychometrics in fMRI). As Vul [2] noted, the reliability of an instrument, including fMRI-based metrics, affects its validity. That is, an instrument’s reliability limits the strength of the associations between that instrument and other measures. Test-retest reliability is an important kind of reliability, particularly for metrics that purport to capture information about traits. There is no universally agreed upon measure of test-retest reliability in fMRI research [3], [4]; however, the intra-class correlation coefficient (ICC) is perhaps the most commonly recommended. Its chief strength is that, like all correlations, it assesses the strength of the association between two measurements. Whereas more standard correlation definitions (e.g., Pearson’s r) are appropriate when the measurements pertain to different constructs (e.g., height and weight), intraclass correlations are appropriate when the two measurements are made regarding the same construct (e.g., repeated measurements separated in time). ICCs reflect the ratio of the between subjects variance to total variance and can be interpreted as reflecting the stability of the measurement between Time 1 and Time 2. Shrout and Fleiss [5] and McGraw and Wong [6] detail several different kinds of ICCs. In fMRI research, it is typical to use a form of the ICC that captures the consistency, or rank ordering of individuals, between Time 1 and Time 2 [3]. Caceres and colleagues describe several methods for calculating this kind of ICC from fMRI data and highlight a method by which voxelwise ICCs are calculated and the median of the voxelwise ICC estimates is used as an index of the reliability of a region.

In a recent, comprehensive review of studies from across the full spectrum of fMRI research, Bennett and Miller [4] noted that no minimum standards have been established for ICCs in fMRI research but that others [7] have proposed the following guidelines in a different research context: Poor (ICC<0.40), Fair (0.40≤ICC<0.60), Good (0.60≤ICC<0.75), and Excellent (ICC≥0.75). Bennett and Miller observed that the average published ICC in fMRI research across designs, psychological tasks/processes, and test-retest intervals was fair (ICC = 0.50), and the majority of studies fell between an ICC of 0.33 and 0.66. They observed that few studies have examined reliability in clinical samples, and that those that have tend to report lower reliability estimates than those using healthy control samples.

### Implications for Longitudinal Treatment Studies

Several studies are currently underway to determine the degree to which various fMRI-derived metrics of structure and function predict, moderate, or mediate response to treatments for psychiatric disorders [8], [9], [10]. An assumption implicit in these investigations is that the neuroimaging markers are themselves relatively stable if left untreated, and that changes observed between one scanning session and the next will be meaningful. The ability to detect the specific effects of a treatment on the functioning of a neural circuit, however, will be affected by the degree to which fMRI-based measures can accurately capture the consistency of the signal when it is consistent. Unless researchers perform multiple scans on individuals prior to the start of treatment, they may not be able to assess the reliability of their metrics. If the fMRI-based metrics in these samples have low test-retest reliability, the statistical tests of the effects of treatment on those measures will likely not have sufficient power [11]. More problematically, when a measure has poor reliability, examining the covariation of that measure with other scales, for example, examining patterns of change over time between fMRI-based and clinical-based measures, can lead researchers to misleadingly conclude that an effect exists when it does not, or vice versa [11], [12].

There are several possible causes for low test-retest reliability estimates when they are observed. One possibility is that estimates of test-retest reliability in patient samples are low because the pattern of functioning of brain regions truly does fluctuate from one testing occasion to the next in these individuals. This could represent random fluctuations in signal across testing sessions or it could reflect natural and important psychological processes, for example, habituation or learning. In either case, it will be difficult to justify the use of the magnitude of the BOLD response as a trait marker of illness. On the other hand, it is also possible that estimates of the reliability of fMRI-based metrics could be affected by the manner in which the data are analyzed. That is, processes like physiological artifacts and motion may introduce structured noise into the observed data, which standard statistical models may not adequately control. If this is the source of low reliability estimates, then different modeling choices might be able to better reveal a higher and more accurate level of consistency that exists in the underlying signal.

### Assumptions of General Linear Model Based fMRI Models

The general linear model (GLM) is ubiquitous in fMRI research, likely due to its relative computational simplicity, its availability in commonly used software packages, and the ease of interpreting its parameter estimates. Although not the only modeling framework available to researchers [13], [14], it is no doubt the most commonly implemented [15]. Full discussion of the specific challenges to the implementation of GLMs with neuroimaging data is beyond the scope of the current work (see [13], [15] for reviews). Instead, we focus on those assumptions of the GLM that can be examined relatively easily with commonly available software. Given the differences between software packages in the implementation of statistical models, and in the available solutions to some of the issues we discuss, and given the popularity of the software package, we focus our work on the implementations available in Statistical Parametric Mapping (SPM) version 8 (SPM8; http://www.fil.ion.ucl.ac.uk/spm).

To examine between subjects effects on brain activity, SPM uses a hierarchical, two-level modeling approach whereby at the first level, separate models are estimated in every voxel separately for every subject. The parameters from these models, either beta weights or contrast weights, are typically examined in second-level, between subjects analyses. As such, it is the reliability of these parameter estimates from the first-level models that are of paramount concern. When model assumptions are met, the estimates from the models are designated best linear unbiased estimates (BLUE; [16]). They are ‘best’ in that they will be the most efficient (i.e., have the smallest standard errors) compared to other estimation frameworks and they are ‘unbiased’ in that they will not systematically over or underestimate the true population value [17]. When model assumptions are not met, the resulting estimates are no longer necessarily BLUE.

In fMRI research, GLMs are intended to be simplified abstractions that can evaluate the extent to which experimental conditions and covariates contributed to the BOLD values that were observed. As such, GLMs make several simplifying assumptions. Although a complete review is beyond the scope of this manuscript [13], [16], [18], one of the primary assumptions holds that the model has been correctly specified in that it is not missing any effects that are known to contribute to the observed scores [16]. Assessing this assumption can be difficult in practice, but one way to examine the extent to which this assumption has been met is to assess the degree to which systematic variation remains in the models’ residuals [19]. That is, examining the degree to which assumptions about the model’s error terms have been met, aside from being a sound statistical practice in general, can help determine whether there are additional systematic effects that could be further removed through model re-specification. Three such assumptions, which can be assessed by examining model residuals, are as follows: 1.) The errors are normally distributed (normality); 2.) The spread or variability in the errors is constant across values of the explanatory variables (homoscedasticity). In the context of fMRI research, this assumption holds that the error variance is constant across every condition in a given scanning protocol [18]; 3.) The errors are assumed to be unrelated to one another (Independence).

Violations of model assumptions can have several different effects. If the model is misspecified in some way, for example, if the hemodynamic response function is not adequately characterized or if non-white physiological noise processes remain in the signal, parameter estimates obtained from the model may become biased [16]. Again, violations of assumptions of normality, homoscedasticity, and independence can indicate such model miss-specification. That is, they can reflect the presence of additional structure in the data, induced by physiological, movement, or other noise processes. If the model does not adequately capture this structure, then beta estimates may be biased. Additionally, although violations of these three assumptions do not themselves directly lead to bias in the beta or contrast estimates, they do affect the standard errors of those estimates, and hence the test-statistics (e.g., t, F) associated with the betas [16], [20]. Additional bias can be introduced when these test-statistics are used to threshold the statistical parametric maps. Finally, the precision of parameter estimates can be compromised when assumptions are violated [19]. If parameter estimates are either biased or made noisier than they ought to due to the modeling approach (and therefore if they are no longer unbiased or efficient estimates of the underlying phenomenon), estimates of test-retest reliability can be affected.

Improving the degree to which a model meets its assumptions does not guarantee an increase in test-retest reliability estimates. If the phenomenon in question is simply not reliable over time, e.g., if it fluctuates over time or if the measurement contains substantial random error, improvement in the fit between model and model assumptions will not necessarily lead to an increase in reliability estimates. Improvement in the model should, however, lead to more precise estimates of the poor reliability that may be present. Stated differently, adopting a modeling approach that more accurately reflects the data (and therefore violates fewer assumptions) should lead to more accurate estimates of test-retest reliability, whether reliability is high or low in the underlying signal.

### The Present Study

In the current study, we examine the degree to which statistical modeling choices can affect estimates of test-retest reliability, as measured by ICCs. In addition, we examine the degree to which misspecifications of the general linear model, as evidenced in voxelwise residual diagnostics, can affect estimates of test-retest reliability in key emotion processing regions across a six-month interval. Numerous previous studies have examined the effects of fMRI pipeline choices on the reproducibility of fMRI results [3], [21]–[29], however the majority of these studies have examined stability either within the same scanning session or during short test-retest intervals (e.g., <2 weeks) over which practice effects may be unlikely to dissipate. Moreover, several early studies used estimates of reliability, such as the percentage of significantly elevated voxels that overlap across testing occasions, which can be affected by arbitrary statistical threshold choices [26]. Additionally, prior work in this area has been conducted almost exclusively using relatively homogenous samples of healthy individuals.

By contrast, in the current study we estimate reliability using voxelwise ICC estimates, and we examine test-retest reliability over a relatively long, six-month interval. One virtue of the use of ICCs as an outcome is that it provides an objective criterion regarding the quality of the modeling framework used to generate the parameter estimates, which can be separated from standard significance testing [28]. ICC estimates are known to be affected by the composition of the sample from which they are calculated [4]. An additional advance in the current study is that we utilize data collected from a heterogeneous sample composed of individuals with no history of any psychiatric disorder as well individuals diagnosed with major depressive disorder and bipolar disorder. This feature of the study should help to maximize the generalizability of the reliability estimates provided. Finally, prior work [30]–[32] has described methods for examining residuals from models of fMRI data to determine the degree to which model assumptions are met. In the current study, we examine whether decisions made during model specification affect the degree to which such assumptions are met and whether they affect the magnitude of the reliability of the model’s parameter estimates. We focus the analysis on those regions of the brain that demonstrate significant task-related activity at Time 1. We expect ICC estimates in these regions to accord with those observed in prior work (ICC ≈0.50). Given the role of the amygdala in processing facial displays of emotion, and given that prior work in our lab using a subset of the data analyzed below observed individual differences in amygdala activity at Time 1 [33]–[35], we also examined test-retest reliability using an anatomical mask of the amygdala. We make no a-priori hypotheses regarding ICC estimates in this region. Finally, we hypothesize that when additional terms are included in GLMs to account for known sources of structured noise, model assumptions will be more closely met and parameter estimates will be more reliable across testing occasions.

## Materials and Methods

In order to examine the relationship between test-retest reliability and the degree to which model assumptions are met, we used data from a sample of healthy control and psychiatric participants, each of whom underwent fMRI scanning during an implicit emotion-processing paradigm on two occasions separated by 6 months. The sample consisted of 48 right-handed, native English-speaking individuals: 17 currently depressed adults diagnosed with major depressive disorder, 15 currently depressed adults diagnosed with bipolar disorder, and 16 healthy control participants. Each participant contributed data from two scanning sessions, resulting in 96 total scans. All participants were followed naturalistically during the six-month assessment window. No specific treatment or intervention was administered in the context of this study, and participants were free to receive, augment, or terminate treatment as needed.

Psychiatric diagnoses were made using the Structured Clinical Interview for Psychiatric Disorders (SCID-P; [36]). The distinction between healthy and psychiatric samples is not the primary focus of this report, and for the purposes of this study, data were pooled for primary analyses. Exclusion criteria were: history of head injury, systemic medical illness, cognitive impairment (score <24 Mini-Mental State Examination; [37]), premorbid IQ estimate <85 (National Adult Reading Test; [38]), standard MRI exclusion criteria (e.g. presence of metallic objects in the body), and having met criteria for an alcohol/substance use disorder within 2 months before the scan (or ever having met criteria for a psychiatric or alcohol/substance use disorder for the healthy control sample). Data from 6 additional subjects, 2 from each group, were excluded either due to poor performance on the task (<75% accuracy) or because of excessive head motion (movement spikes >3 mm).

### Ethics Statement

The University of Pittsburgh Institutional Review Board (IRB) approved the study protocol. The IRB approved all procedures for acquiring informed consent, recognizing that individuals with psychiatric diagnoses may, under certain circumstances, be considered vulnerable populations. The protocol called for any potential participant who was deemed by the trained study clinicians to be a danger to themselves or others or who was deemed to be incapable of caring for themselves, and thus of providing informed consent, to be referred for emergency treatment. No treatment was initiated, delayed, stopped, or in any way altered in order to facilitate or effect participation in this study. Participants were expressly informed that their decision to participate or not in this study would in no way affect their ability to receive current or future treatment at this institution. After complete description of the study, all individuals who participated in this study provided written informed consent. All data utilized in the present analyses were stored and analyzed using subject ID numbers only.

### Paradigm

Participants completed a 12.5-minute emotional dynamic faces task during fMRI. Stimuli comprised faces from the NimStim set [39] that were morphed in 5% increments, from neutral (0% emotion) to 100% emotion for 4 emotions: happy, sad, angry and fear [33], [40]–[42]. Morphed faces were collated into 1 s movies progressing from 0% to 100% emotional display. In control trials, movies comprised a simple shape (dark oval) superimposed on a light-grey oval, with similar structural characteristics to the face stimuli, which subsequently morphed into a larger shape, approximating the movement of the morphed faces. Separate control trials, not examined below, were also presented whereby neutral faces morphed from one identity to a different identity. There were three blocks for each of the four emotional conditions, with twelve stimuli per block, and six shape-control blocks with six stimuli per block. Emotional and control blocks were presented in a pseudorandomized order so that no two blocks of any condition were presented sequentially. Participants were asked to use one of three fingers to press a button indicating the color of a semi-transparent foreground color flash (orange, blue, or yellow) that appeared during the mid 200 ms-650 ms of the 1 s presentation of the dynamically-changing face. The emotional faces were task-irrelevant and, thus, were processed implicitly.

### Data acquisition

Neuroimaging data were collected using a 3.0 Tesla Siemens Trio MRI scanner at the Magnetic Resonance Research Center in the University of Pittsburgh Medical Center. Structural 3D axial MPRAGE images were acquired in the same session (TR/TE = 2200/3.29 ms; Flip angle 9°, FOV: 256×192 mm^2^; Slice thickness: 1 mm; Matrix: 256×256; 192 continuous slices). BOLD images were then acquired with a gradient echo EPI sequence during approximately thirteen minutes (378 successive brain volumes) covering 39 axial slices (3.2 mm thick; TR/TE = 2000/28 ms/ms; FOV = 205×205 mm^2^; matrix = 64×64; Flip angle 90°).

### Functional Neuroimaging Data Processing: Default Pipeline

Data were preprocessed and analyzed with statistical parametric mapping software (SPM8; http://www.fil.ion.ucl.ac.uk/spm). For all modeling pipelines described below, the preprocessing steps were held constant. During preprocessing, images were realigned, using the default linear, 6-parameter, rigid body transformation implemented in SPM8. Anatomical images were corregistered to the mean functional images. Images were spatially normalized into Montreal Neurological Institute [MNI] space, resampled to 3×3×3 mm^3^, and spatially smoothed with an 8-mm full-width at half maximum (FWHM) Gaussian kernel.

In our default modeling pipeline, the experiment was modeled as a block design and first-level fixed-effect GLMs were fit. The SPM8 default settings were used, such that no global signal normalization was employed, and the default double gamma hemodynamic response function was assumed. Restricted maximum likelihood models are used by SPM to estimate the degree of serial correlation in the data during first level model estimation, assuming a global first order autoregressive plus white noise structure [43]. The data are transformed using the resulting estimates, and generalized linear models are fit [43]. This procedure aims to remove first-order autocorrelations present in the residuals, thereby helping to address possible violations of the independence assumption. The removal of autocorrelations at longer lags is accomplished by the use of a high-pass temporal filter [43]. We used the default high-pass filter cutoff of 128 seconds for all pipelines. Each of the four emotion conditions (anger, fear, sad, happy) were entered as separate conditions in the design matrix, as was the shape condition, which served as the baseline. For all results reported below, the contrast of interest was that comprised by all emotional conditions minus shapes. Movement parameters from the preprocessing procedure were entered as covariates of no interest to control for subject movement. Data from each testing occasion were modeled separately.

### Functional Neuroimaging Data Processing: Alternative Pipelines

We examined the degree to which two changes to the model specification could affect residual diagnostics and ICC estimates: 1) We added flexibility to the hemodynamic response function that we assumed (Flexible HRF); and 2) we added additional corrections for fluctuations in the global signal (mCompCor). These two changes were selected over other approaches, e.g., nonparametric models, because they are each relatively easy to implement through the addition of extra columns in the design matrix. In order to model the hemodynamic response function more flexibly, we included both temporal and dispersion derivatives of the HRF in the model as additional basis functions. Temporal derivatives allow for small individual differences in the timing of the peak response, whereas dispersion derivatives allow for small differences in the width of the HRF. In order to do so, we treated the experiment as a mixed block/event-related design at the first level. In order to add additional corrections for potential confounds in the global signal, we used a method inspired by the CompCor technique of Behzadi and colleagues [44], [45]. This method is based on the assumption that regions of white matter, cerebrospinal fluid (CSF) and voxels with high standard deviation are those most contaminated with physiological noise, and thus estimates of signal fluctuations from these regions make effective regressors in order to correct for physiological noise. We simplified the approach relative to previous instantiations, so as to reduce variation across individuals regarding the noise component that is regressed out as well as to use as few regressors as possible in order to keep the model as simple as possible. Here, we pooled the time-series of voxels with a high temporal standard deviation (top 2% of the whole brain) with the time-series of voxels located within a white matter and CSF mask and computed the mean, leading to a single time-series we refer to as a modified CompCor regressor (mCompCor, see Methods S1 for additional detail). This array was included as a nuisance variable in our first level analyses, which treated the data as a block design, as did the default pipeline, in order to maximize design efficiency when possible. Finally, we used a pipeline that combined both the Flexible HRF and mCompCor pipelines. Due to the inclusion of the additional basis functions to more flexibly model the HRF, data were modeled as a mixed block/even design in the combined approach.

### Regions of Interest

We focused our analyses of test retest reliability on those regions showing significant task-related activity during the first testing occasion (whole brain, FWE cluster corrected at p<.05, with a cluster forming threshold of p<0.001), because we wanted to understand the effects of modeling choices on test-retest reliability in those regions most closely associated with task activity at time 1. In addition, we examined test-retest reliability using an anatomical, bilateral amygdala mask, as defined by the Wake Forest Toolbox PickAtlas Talairach Daemon template [46]. Prior work in our laboratory has observed abnormal amygdala activity in response to this task in individuals with bipolar disorder [33], major depressive disorder [34], [35], and in combat veterans with elevated symptoms of post-traumatic stress [47]. The reports of activity in participants with bipolar disorder and major depressive disorder [33]–[35] used a subsample of the data examined below, but were focused on different topics (individual differences and group differences in amygdala activity to specific emotions at Time 1). No study in our laboratory has examined response to this task across time.

### Outcomes of interest

#### Intraclass Correlation Coefficients (ICC)

Following Caceres and colleagues [3], and using elements of the ICC toolbox they describe, we calculated ICC estimates separately for each voxel using formula ICC (3,1), described by Shrout and Fleiss [5]. This ICC statistic is calculated from the results of a two-way ANOVA model in which subjects are treated as random effects and sessions are treated as fixed effects. This form of the ICC measures the consistency between the repeated measurements, not the absolute agreement between them. Like the results of Caceres and colleagues, the choice of other ICC calculations, e.g., ICC3 (a,1) [6], did not appreciably change the results reported below. Following Caceres, we report the median of the voxel-wise ICCs (mvICC) in a particular region as our primary reliability statistic of interest. Because the four pipelines represent repeated measures, conducted within each voxel, and because distributions of test statistics such as ICCs are not necessarily normal, tests between pipelines were conducted using Freidman’s test, a non-paramteric test of ranked data akin to a one-way analysis of variance for repeated data. The statistics of interest and the corresponding p-values were estimated using SAS PROC FREQ [48]. Post-hoc tests were conducted using Nemenyi tests [49], which have similar properties to Tukey tests in the context of standard ANOVAS and correct for multiple comparisons [50]. Nemenyi tests were calculated using the % Nemenyi SAS macro [50].

#### Examination of Model Residuals

Based in part on the recommendations by Luo and Nichols [30], we examined the following indices of model residuals, separately for each voxel in the ROIs: The normality assumption was assessed using the Shapiro-Wilk test, which tests the null-hypothesis that the residuals are normally distributed. Homoscedasticity was examined using the Breusch-Pagan test, which examines whether the variance of the residuals is independent of the design matrix. The Durbin-Watson test was used to examine the independence assumption. It examines whether a first-order autocorrelation pattern is present in the residuals (see Methods S1 for additional detail regarding analysis of the model residuals). Because the data regarding assumption failures had a nested structure (e.g., voxels nested within participants), we used generalized linear mixed effects models, implemented in SAS PROC GLIMMIX, to examine differences among pipelines in the degree to which GLM model assumptions were violated. The dependent variable was a binary indicator representing whether the assumption in question was violated or not (as determined by a significant p-value from the assumption test in question).

## Results

### Neural Activity at Times 1 and 2

**Figure 1:**
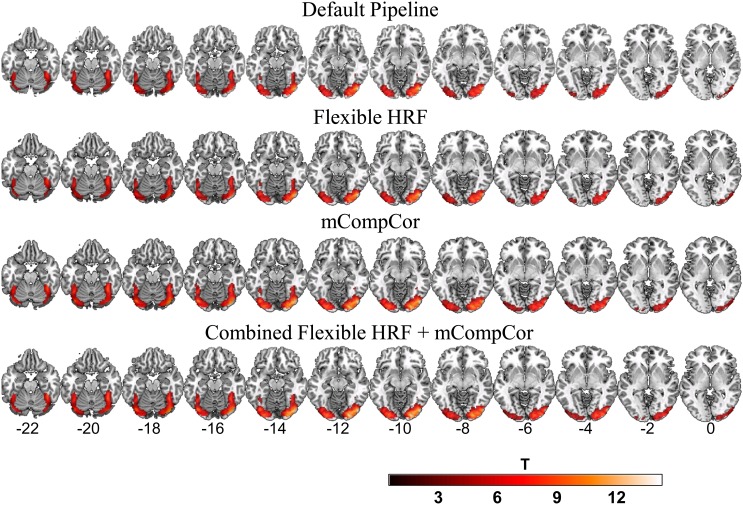
Task Related Neural Activity at Time 1. Plotted values represent t-statistics from the emotional-faces-minus-shapes contrast during the first testing occasion. Default pipeline: data were modeled as a block-design with a canonical hemodynamic response function (HRF); Flexible HRF pipeline: data were modeled as a mixed block/event design and temporal and dispersion derivatives were added to the design matrix as additional basis functions; mCompCor pipeline: data were modeled as a block design and a additional regressor was added to the design matrix to account for physiological noise in the global signal; Combined Flexible HRF and mCompCor pipeline: data were modeled as a mixed block/event design and both the Flexible HRF and mCompCor components were implemented.

Figure 1 displays BOLD response (p<.05, FWE corrected) to the dynamically changing emotional faces, minus the dynamic shape baseline condition, for all four pipelines. In each case, large clusters of activity were observed in bilateral visual regions extending anteriorly to include portions of the fusiform gyrus. The smallest clusters of activity were observed for the default pipeline (total number of significant voxels = 999) and the flexible-HRF model (total number of significant voxels = 994), whereas the mCompCor and the combined alternative pipelines demonstrated the largest cluster extents (total number of significant voxels = 1258 and 1248, respectively).

Because each pipeline yielded a different number of activated voxels, in order to make comparisons among the pipelines, a mask was created that represented the regions of overlap (in bilateral visual and fusiform regions) among the four pipelines. Using this mask, the nonparametric, repeated measures Friedman test was performed on the voxel-wise t-statistics. It revealed that the t-test statistic distributions differed among the four pipelines (Chi-Square (3) = 2085.87, p<0.001). Post-hoc Nemenyi tests revealed that both the combined alternative pipeline and the mCompCor pipeline generated larger t values compared to the default and the flexible HRF pipelines (all Chi-Squares (3) ≥204.88, *p*s<0.001). The combined alternative and mCompCor pipelines did not differ from each other (Chi-Square (3) = 0.29, *p* = 0.96), nor did the default pipeline and the flexible HRF pipeline (Chi-Square (3) = 0.64, *p* = 0.89).

**Figure 2:**
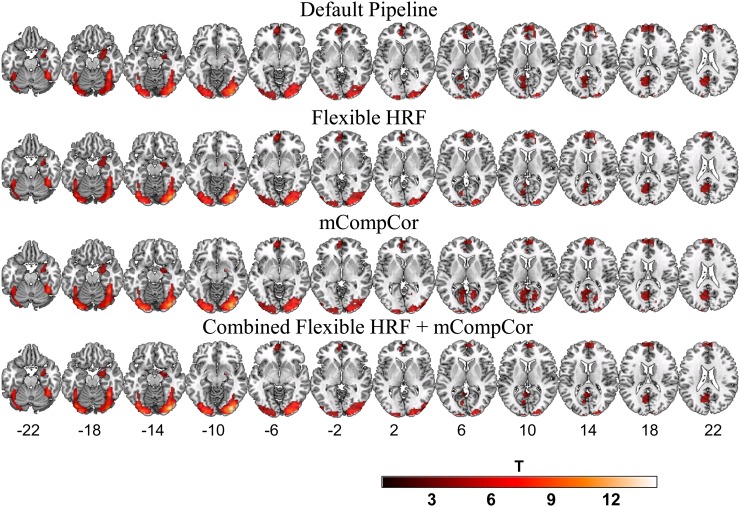
Task Related Neural Activity at Time 2. Plotted values represent t-statistics from the emotional-faces-minus-shapes contrast during the second testing occasion. Default pipeline: data were modeled as a block-design with a canonical hemodynamic response function (HRF); Flexible HRF pipeline: data were modeled as a mixed block/event design and temporal and dispersion derivatives were added to the design matrix as additional basis functions; mCompCor pipeline: data were modeled as a block design and a additional regressor was added to the design matrix to account for physiological noise in the global signal; Combined Flexible HRF and mCompCor pipeline: data were modeled as a mixed block/event design and both the Flexible HRF and mCompCor components were implemented.

Figure 2 displays BOLD response (p<.05, FWE corrected) on the second imaging occasion, six-months later. Again, robust activity in bilateral visual regions extending to the fusiform gyrus was observed in all four pipelines. In addition, activity was also observed in several regions involved in the default-mode network, including regions in the dorsal and ventral medial prefrontal cortex, left parietal regions including the cuneus and precuneus extending to the posterior cingulate (supra-threshold activity in right-parietal regions was also observed in the mCompCor only pipeline), and right limbic regions including portions of the amygdala, hippocampus and parahippocampal gyrus.

### Test-Retest Reliability

**Figure 3:**
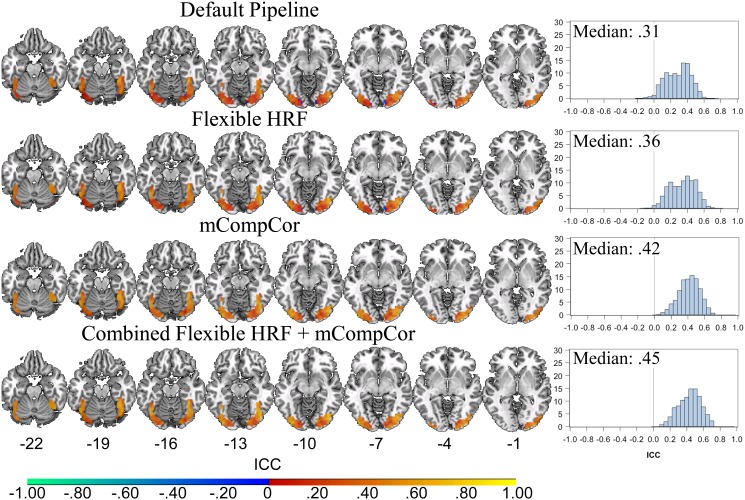
Voxelwise ICC Estimates in Task Related Regions at Time 1 in all Pipelines. Plotted values on the left represent voxelwise intraclass correlation coefficients (ICCs) in the regions that showed overlapping activity between the four pipelines at time 1. Plots on the right represent histograms of the voxelwise ICCs from the overlap mask, along with the Median ICC for the region. Default pipeline: data were modeled as a block-design with a canonical hemodynamic response function (HRF); Flexible HRF pipeline: data were modeled as a mixed block/event design and temporal and dispersion derivatives were added to the design matrix as additional basis functions; mCompCor pipeline: data were modeled as a block design and a additional regressor was added to the design matrix to account for physiological noise in the global signal; Combined Flexible HRF and mCompCor pipeline: data were modeled as a mixed block/event design and both the Flexible HRF and mCompCor components were implemented.

The voxel-wise ICC estimates of six-month test-retest reliability in visual regions, using the mask of overlapping activity at Time 1 described above, differed among the four pipelines (Friedman’s test: Chi-Square (3) = 1587.22, *p*<0.001). Figure 3 displays voxelwise ICC estimates of the six-month test-retest reliability in regions demonstrating task related activity, as well as median voxelwise ICCs for each pipeline. Nemenyi post-hoc tests revealed that the ICCs associated with each pipeline differed significantly from each other pipeline (all Chi-squares (3) >46.34 all *ps*<0.001) with one exception. ICCs estimated in the combined alternative pipeline and the mCompCor pipeline did not differ (Chi-square (3) = 5.98, p = 0.11). Thus, the Median ICC increased from the default pipeline, mvICC = 0.31 (SE = 0.01), to the flexible-HRF only model, mvICC = 0.36 (SE = 0.01), and finally to the mCompCor only, mvICC = 0.42 (SE = 0.01), and the combined alternative, mvICC = 0.45 (SE = 0.01) models. In secondary analyses, the ICCs estimated separately for each group of participants likewise improved with increased pipeline complexity (Table S1).

**Figure 4:**
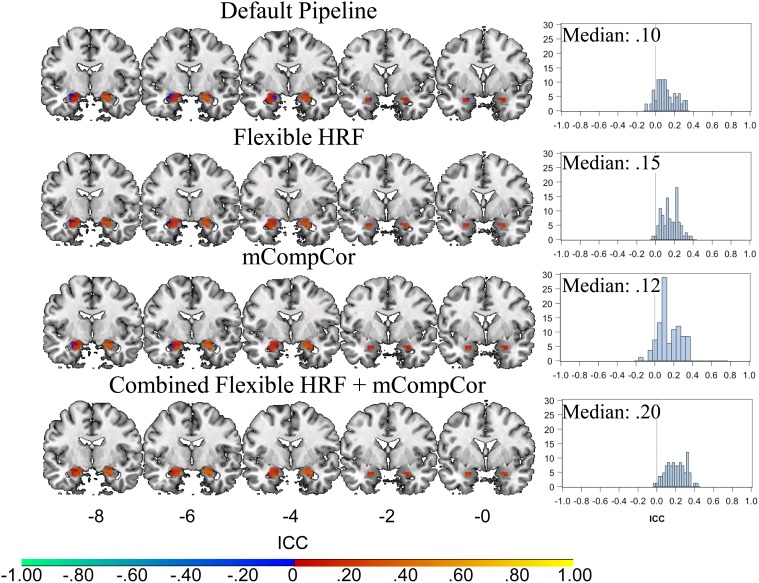
Voxelwise ICC Estimates in Bilateral Amygdala. Plotted values on the left represent voxelwise intraclass correlation coefficients (ICCs) in bilateral amygdala. Plots on the right represent histograms of the voxelwise ICCs from the bilateral amygdala mask, along with the Median ICC for the region. Default pipeline: data were modeled as a block-design with a canonical hemodynamic response function (HRF); Flexible HRF pipeline: data were modeled as a mixed block/event design and temporal and dispersion derivatives were added to the design matrix as additional basis functions; mCompCor pipeline: data were modeled as a block design and a additional regressor was added to the design matrix to account for physiological noise in the global signal; Combined Flexible HRF and mCompCor pipeline: data were modeled as a mixed block/event design and both the Flexible HRF and mCompCor components were implemented.

Test-retest reliability was also examined for all four pipelines using a bilateral amygdala mask. As displayed in Figure 4, the distributions of ICC estimates across the full sample were consistently smaller in the amygdala compared to those reported above, and all fell in the poor range. Freidman’s test indicated that the distributions of ICC values in bilateral amygdala differed among the four pipelines (Chi-Square (3) = 127.67, *p*<0.001). Post-hoc Nemenyi tests revealed that the combined alternative pipeline had higher ICCs compared to the default pipeline (Chi-Square (3) = 29.00, *p*<0.001) and the mCompCor pipeline (Chi-Square (3) = 9.75, *p = *0.02). The flexible HRF model also demonstrated higher ICCs than the default pipeline (Chi-Square (3) = 8.22, *p* = 0.04). No other comparisons were significant (all Chi-squares (3) <6.35, *ps*>.09. Secondary analyses (Table S1) revealed that ICCs estimates improved between the default and combined alternative pipelines for the participants diagnosed with major depressive disorder (mvICC = 0.20, SE = 0.03 for the combined pipeline) and for those with bipolar disorder (mvICC = 0.43, SE = 0.02, for the combined pipeline, which falls in the fair range). No improvement was observed for the healthy control participants (mvICC = 0.04, SE = 0.06, for the combined pipeline).

### Model Assumptions

Table 1 displays indices of the degree to which modeling assumptions were met by each pipeline in the overlapping regions of task-related activity at Time 1. Generalized linear mixed effects models revealed that the pipelines differed with regard to the proportion of voxels meeting each of the three assumptions (all *F*s (3, 141) >358.50, *p*s<0.001). The combined alternative model demonstrated the lowest proportion of violations of assumptions across the 87,744 GLM models (48 participants×2 assessments×914 voxels in the ROI) compared to the other pipelines (all *t*s (141) >17.55 *p*s<0.001), with two exceptions: the combined alternative pipeline did not differ from the mCompCor pipeline with respect to the assumption of normality (*t*(141) = 1.69, *p* = 0.09), and the mCompCor only pipeline demonstrated fewer violations of the independence of errors assumption than did the combined alternative pipeline (*t*(141) = −35.95, *p*<0.001).

**Table 1:** Proportion of Models for which the Assumption Test Failed.

Pipeline	Independence	Normality	Homoscedasticity
	Bilateral Visual^a^		
Default	50.3%	41.8%	47.0%
Flexible HRF	57.9%	40.8%	37.3%
mCompCor^b^	38.8%***	37.3%***	42.1%
Combined HRF + mCompCor^b^	46.4%	36.9%***	34.1%***
	**Bilateral Amygdala** c		
Default	63.4%	38.9%	37.6%
Flexible HRF	52.3%	37.8%	30.4%
mCompCor	48.7%	33.1%	33.4%
Combined HRF + mCompCor	38.0%***	31.5%**	28.8%**

Pipeline(s) with the fewest failures compared to each of the other pipelines: ** at *p*<0.01; *** at *p*<0.001.

a Number of models = 87744 per pipeline. Generalized linear mixed effects models indicated that the pipelines differed with regard to the proportion of models meeting each assumption (all *F*s (3, 141) >358.50, *p*s<0.001).

b The mCompCor and the Combined HRF + mCompCor models did not differ at p<0.05 with respect to the normality assumption. Both are indicated as representing fewer model failures than the remaining pipelines.

c Number of models = 8448 per pipeline. The pipelines differed with regard to the proportion of models meeting each assumption (all *F*s (3, 141) >82.65, *p*s<0.001).

Regarding the bilateral amygdala regions, like the results of the visual regions, the pipelines differed from one another with regard to the proportion of the 8,448 models (48 participants×2 assessments×88 voxels in the ROI) in which each of the three model assumptions were met (all *F*s (3, 141) >82.65, *p*s<0.001). The combined alternative model demonstrated the lowest proportion of assumption violations compared to the other pipelines across all assumption tests (all *t*s(141) >2.74, *p*s<0.01).

## Discussion

Establishing the test-retest reliability of fMRI markers is of paramount importance if such metrics are to be used in the future to help guide diagnosis and treatment of psychiatric conditions. The results of the current investigation demonstrate that the reliability of fMRI-derived estimates can differ substantially depending on the details of the modeling approach used to conduct the analysis. Indeed, we observed an improvement in test-retest reliability estimates of approximately 45% between the default and the full alternative modeling approach. That is, adding additional terms to the first level models in order to better account for the hemodynamic response function and for physiological artifacts in the global signal largely resulted in higher test-retest reliability estimates. In visual processing regions, this result was observed both in the full sample as well as in each subgroup, suggesting that improvements to the statistical models resolved within-subject variability in each of the groups of patients as well as across the sample as a whole. To the extent that the added terms resulted in models that better captured the processes that generated the observed BOLD signal data, it is not surprising that parameter estimates regarding task effects were more likely to be BLUE. What is potentially more surprising, and is a result that was not necessarily guaranteed, is that such parameter estimates were revealed to be more stable across testing sessions. The omission of important independent variables in GLMs can result in biased and imprecise parameter estimates, likely rendering it more difficult to observe consistent estimates from two models of data separated in time. The inclusion of the additional terms in the design matrix also resulted in fewer assumption violations, suggesting that the inclusion of these terms resulted in the removal of additional structured noise processes.

The six-month test-retest reliability of fMRI markers associated with processing emotional faces fell in the fair range in visual regions using the two pipelines that best met the modeling assumptions and were quite close in magnitude to the mean reported by Bennett and colleagues from fMRI studies across the literature. These findings suggest that activity in these regions would provide an adequate target in future work using this (or perhaps a similar) paradigm to examine change as a consequence of an experimental or treatment manipulation. By contrast, stability of the signal was poor in the bilateral amygdala across the full sample. For healthy control participants, ICC estimates were near zero in the amygdala and did not appear to be affected by pipeline choices. However, for individuals with bipolar disorder, ICC estimates fell in the fair range and were affected by statistical modeling choice. There are several possible reasons for these findings. It may be that amygdala activity during emotion processing is a more trait-like feature of bipolar disorder, whereas a natural amygdalar habituation process may have operated for the healthy control participants. More work will be needed to test these possibilities. If such a finding were confirmed, it would suggest that groups can differ in the degree to which signal in a particular region is stable across assessments. Moreover, the fact that modeling choices affected ICC estimates in the amygdala for the psychiatric sample but not the healthy control sample suggests that although stability of fMRI signal is not guaranteed for any sample even with improvements to the modeling strategy, using modeling tools that better account for sources of within subject variability can help to reveal intersession stability when it is present.

It is not entirely clear from the results of this study why additional activity was observed at the second testing occasion in several regions involved in default mode processing [51], as well as in portions of the amygdala. Future work in this laboratory will examine whether this pattern was universally observed, or like the stability effects in the amygdala, whether there are meaningful individual (or group) differences with regard to the increase in default mode activity at the second testing occasion. It is possible that instability in activity across testing occasions reflected a meaningful process (e.g., learning, strategy changes, habituation, practice effects), at least in some groups. Should such a hypothesis be supported by the data [52], it would imply that probing the system during the first testing occasion may have changed it in a meaningful way. Assessing test-retest stability is only sensible when one has a reasonable expectation that the construct to be measured should be the same across the time points. If the first testing occasion changed the processing in key regions in the brain, it may not be sensible to expect signal at subsequent testing occasions to be consistent with that observed at the first, regardless of the degree to which one’s models are correctly specified.

In this work, we used two alternative specifications of the first-level models that are relatively straightforward to implement through the inclusion of additional terms in the design matrix. We use these as examples to examine whether modeling choices affect test-retest reliability estimates. We do not intend to imply that these particular-modeling steps should be universally adopted across all designs and tasks. Indeed, in certain cases in the present study, e.g. the inclusion of additional basis functions to more flexibly model the HRF, an increase in error-independence violations was observed in visual regions. This likely reflects a complex relationship between autocorrelation processes and HRF model parameterizations, which may differ across regions in the brain. Furthermore, researchers are faced with a critical decision regarding the trade-off between model complexity and interpretability when deciding how many parameters to include in a statistical model (as well as whether or not to assume that the same model is appropriate across all portions of the brain). In this paper, we opted to include a relatively small number of added parameters in order to assess the affects of relatively minor model re-specifications. The key finding was that these choices can have a profound impact on the ability to recover parameter estimates from one scanning session to another. These considerations are particularly important for longitudinal treatment studies in which an experimental intervention is introduced between scans. In many such studies, resource constraints may not allow for the collection of multiple baseline assessments in order to examine test-retest reliability directly. In such cases, it may be all the more important to ensure that the pipeline that most adequately meets model assumptions is used.

One of the statistical modeling choices we examined was the addition of a regressor designed to account for physiological noise, which we derived from elements of the global signal. The use of global signal corrections in fMRI processing is somewhat controversial. The goal of such an approach is to remove additional structured noise, typically thought to reflect physiological processes, from the data. Early implementations of global signal correction included the use of proportional scaling to correct for global signal inhomogenieties. More recently, Behzadi and colleagues described techniques [44] for generating regressors to account for physiological noise processes using signal observed in white matter, CSF, as well as voxels displaying high temporal variability. Chai and colleagues [45] present evidence that, at least with respect to resting-state data, this newer approach may avoid some of the limitations of the earlier approaches, such as a reduction in sensitivity and the possible exaggeration of patterns of deactivation and anticorrelation [53]–[56]. We modified and simplified the approach described by Behzadi and colleagues, and observed improvements in test-retest reliability when the approach was used.

### Limitations

The conclusions that can be drawn from the current work must be understood in the context of several limitations. First, the ICC represents a statistical estimate of test-retest reliability. It is not a direct indicator of the true test-retest stability that exists in the underlying raw signal. Given the manner in which it is calculated, ICC values can be affected by factors other than the true underlying stability of the signal. Because ICCs are proportional to the between subjects variability, homogeneous samples can lead to reduced ICC estimates [4]. It is not clear, however, that other measures of test-retest stability have superior properties compared to the ICC. We believe that one strength of the current report is the inclusion of a heterogeneous sample composed of both patients diagnosed with mood disorders as well as individuals with no psychiatric illnesses. Moreover, secondary analyses in visual regions revealed that changes to the statistical analyses pipelines improved ICC estimates for each of the subgroups. As such, any sample homogeneity present in the current work is unlikely to account for the pattern of results whereby changes to the modeling framework affected ICC estimates.

Second, we did not examine the effect of alternative preprocessing steps or processing decisions such as movement thresholds on ICC estimates, in part because others have previously commented on these issues [3], [24], [28], [29], and we limited the scope of the current work to GLM analysis of fMRI data with SPM software. Software packages may differ in the degree to which the default options generate models that conform to model assumptions. Indeed, the most popular software programs differ from one another in the manner in which temporal autocorrelations in the data are estimated and modeled [57], and each program offers users different ways to flexibility alter their statistical pipelines. A complete comparison of the differences between software packages is beyond the scope of this work. The crucial finding is that regardless of the choice of software, the best choice among fMRI analysis pipelines is the one that best describes the data, best accounts for noise processes, and best meets the relevant statistical assumptions, given the properties of the data. Similarly, the results described in the current work are most relevant to general linear modeling frameworks. Other statistical approaches for fMRI data make different assumptions, however, a common assumption across most statistical models is that the model in question has adequately captured the relevant processes responsible for generating the data. As such, procedures like more flexibly modeling the HRF and accounting for physiological noise would likely improve test-retest reliability estimates from non-GLM based models as well.

Third, the time delay between the two scanning sessions, six months, was relatively long. Shorter intervals may have yielded stronger test-retest effects. Additional work will be needed in order to fully describe test-retest reliability estimates at different time intervals, as well as the psychological processes that may affect changes in signal over time. Finally, in order to limit the scope of the study, we focused only on the amygdala and on those regions demonstrating task related activity at Time 1, using a relatively strict threshold. It is possible that other regions in the brain demonstrated better (or worse) ICC estimates.

### Conclusions

This is the first report of which we are aware to examine the relationship between the reliability of task-based fMRI assessments separated by a long test-retest interval and the adequacy of the statistical models used to analyze the data. Establishing and optimizing the test-retest reliability of fMRI-based metrics will be critical if these measures are to be useful clinically. There is no doubt that much additional work is needed before the field will reach a consensus on the optimal statistical modeling strategies for analyzing data from particular neuroimaging paradigms. The results of the current study suggest that these modeling choices will affect estimates of the reliability of fMRI-based metrics, and by extension, the conclusions that may obtain in longitudinal and treatment studies.

## Supporting Information

Table S1 **Median voxelwise ICC estimates for each modeling pipeline in each participant group.**
(DOCX)

Methods S1 **Supplemental information regarding the generation of the mCompCor regressor and the analysis of model residuals.**
(DOCX)
