# Supplementary material for: Model Specification and the Reliability of fMRI Results: Implications for Longitudinal Neuroimaging Studies in Psychiatry
Source: PLoS One. 2014 Aug 28;9(8):e105169. doi: 10.1371/journal.pone.0105169 (PMC4148299; doi:10.1371/journal.pone.0105169)
Supplement: Table S1 — Median voxelwise ICC estimates for each modeling pipeline in each participant group. (DOCX) [file pone.0105169.s001.docx]

| Table S1. Median Voxelwise ICC Estimates for each Pipeline in each Group | | | |
| --- | --- | --- | --- |
| Pipeline | MDD | HC | BD |
|  | Bilateral Visual | | |
| Default | 0.26 (01) | 0.20 (01) | 0.33 (01) |
| Flexible HRF | 0.29 (01) | 0.29 (01) | 0.39 (01) |
| mCompCor | 0.46 (01) | 0.34 (01) | 0.41 (01) |
| Combined HRF + mCompCor | 0.47 (01) | 0.38 (01) | 0.43 (01) |
|  | Bilateral Amygdala | | |
| Default | -0.03 (0.04) | 0.09 (0.03) | 0.35 (0.03) |
| Flexible HRF | 0.01 (0.03) | 0.06 (0.05) | 0.39 (0.02) |
| mCompCor | 0.15 (0.03) | 0.07 (0.04) | 0.36 (0.04) |
| Combined HRF + mCompCor | 0.20 (0.03) | 0.04 (0.06) | 0.43 (0.02) |
| Values are median voxelwise ICC estimates, calculated separately for each group for each pipeline. Parenthetical values represent standard errors. MDD = Participants diagnosed with major depressive disorder; HC = Healthy control participants; BD = Participants diagnosed with bipolar disorder | | | |
