# Supplementary material for: Model Specification and the Reliability of fMRI Results: Implications for Longitudinal Neuroimaging Studies in Psychiatry
Source: PLoS One. 2014 Aug 28;9(8):e105169. doi: 10.1371/journal.pone.0105169 (PMC4148299; doi:10.1371/journal.pone.0105169)
Supplement: Methods S1 — Supplemental information regarding the generation of the mCompCor regressor and the analysis of model residuals. (DOCX) [file pone.0105169.s002.docx]

Methods Supplement.

Additional technical details are provided below regarding the generation of the mCompCor regressor and the analyses of model residuals.

Generating the mCompCor Regressor:

In order to calculate the modified CompCor (mCompCor) regressor, individualized white matter (WM) and cerebrospinal fluid (CSF) masks were first generated for each subject. An initial WM/CSF mask was generated by segmenting the MNI template and applying partial volume thresholds to avoid regressing out smoothed signal from gray matter (B. Patenaude & A. Etkin, personal communication). We resampled this WM/CSF image to match the dimensions of the images used in the present study, and masked the image separately for each subject using the individual brain-mask files generated by SPM. Signal was extracted from each voxel using the individualized WM/CSF mask for each subject.

Next, normalized but non-smoothed preprocessed functional images were skull-stripped, timeseries data were extracted from each voxel, and temporal standard deviations were calculated. Voxels representing the top 2% of temporal standard deviations across the brain were identified.

Timeseries from voxels in the individualized WM/CSF masks were combined with timeseries from the voxels with high temporal standard deviations, and quality assurance checks were run to ensure that no voxel was counted twice. The mCompCor variable was calculated as the mean timeseries across all voxels identified above. In preliminary work we observed that generating one regressor representing the mean across all identified voxels versus generating two regressors, one for voxels in the WM/CSF mask and one for those with high temporal standard deviations, yielded similar results. We opted to use the mean across all identified voxels in order to reduce the number of parameters in the GLM model.

Examining Model Residuals:

In order to examine first level model residuals in SPM8, we first modified the spm_spm.m code by commenting out those sections that delete the residual files generated by SPM during first level model estimation (see <http://akiraoconnor.org/2010/03/24/keeping-residuals-after-spm-estimation/>). Residual timeseries were extracted from each voxel within each region of interest. For each voxel, we calculated the Shapiro-Wilk test using the MATLAB function swtest (available at [www.mathworks.com](http://www.mathworks.com/)), the Dubin-Watson test using the MATLAB function dwtest (available in the Statistics Toolbox), and the Breusch-Pagan test using the MATLAB function TestHet (available at [www.mathworks.com](http://www.mathworks.com/)).
